#  Risk factors for colistin-resistant Enterobacteriaceae in a low-endemicity setting for carbapenem resistance – a matched case–control study

**DOI:** 10.2807/1560-7917.ES.2018.23.30.1700777

**Published:** 2018-07-26

**Authors:** Andrea C Büchler, Christian Gehringer, Andreas F Widmer, Adrian Egli, Sarah Tschudin-Sutter

**Affiliations:** 1 Division of Infectious Diseases & Hospital Epidemiology, University Hospital Basel, University of Basel, Basel, Switzerland; 2 Division of Internal Medicine, University Hospital Basel, University of Basel, Basel, Switzerland; 3 Division of Clinical Microbiology, University Hospital Basel, University of Basel, Basel, Switzerland; 4 Applied Microbiology Research, Department of Biomedicine, University of Basel, Basel, Switzerland; 5 Department of Clinical Research, University Hospital Basel, University of Basel, Basel, Switzerland

**Keywords:** colistin resistance, Enterobacteriaceae, risk factor, carbapenem resistance, low-endemicity setting

## Abstract

Emergence of colistin resistance has been related to increased use in clinical settings, following global spread of carbapenem-resistant Gram-negative bacteria. Use of colistin in animal production may constitute a further source of spread of resistant strains to humans. We sought to determine risk factors for human colonisation or infection with colistin-resistant *Escherichia coli* and *Klebsiella pneumoniae* in a setting where colistin is mainly used for animal production. **Methods:** This retrospective matched case–control study was performed during a 5-year period at two university-affiliated hospitals in Basel, Switzerland. Conditional univariable logistic regression was used to calculate odds ratios (OR) for colistin resistance. All variables found to be significant in univariable analyses were included in the conditional multivariable regression model using stepwise forward and backward selection. **Results:** Forty-two cases (33 with colistin-resistant *E. coli*, 9 with colistin-resistant *K. pneumoniae*) and 126 matched controls were identified. Baseline characteristics, comorbidities, prior exposure to antibiotics and healthcare settings did not differ between cases and controls, except for prior exposure to carbapenems, hospitalisation and stay abroad during the prior 3 months. In multivariable analyses, only prior exposure to carbapenems remained associated with colistin resistance (OR: 5.00; 95% confidence interval (95% CI): 1.19–20.92; p = 0.028). **Conclusion:** In a low-endemicity setting for carbapenem resistance, prior exposure to carbapenems was the only risk factor for colonisation or infection with colistin-resistant *E. coli* or *K. pneumoniae*. Prior exposure to colistin was not significantly associated with detection of colistin resistance, which mainly occurred in the absence of concurrent carbapenem resistance.

## Introduction

New resistance mechanisms against antibiotics are emerging worldwide. During the past decade, resistance against broad-spectrum antibiotics has further increased, especially in bacteria belonging to the Enterobacteriaceae. The rapid increase of enzymes conferring resistance to beta-lactam antibiotics, such as extended spectrum beta-lactamase (ESBL), *Klebsiella pneumoniae* carbapenemases (KPC) and New Delhi metallo-beta-lactamase (NDM) are causing serious concern [1] as the remaining treatment options are limited, thus fostering the increased use of antibiotics of last resort, such as colistin [2].

Colistin belongs to the family of polymyxins with broad-spectrum activity against Gram-negative bacteria. Until November 2015, colistin resistance was only known to be caused by chromosomal mutation, but recent reports from Asia, Europe and the Americas have documented that genes conferring colistin resistance (i.e. *mcr*-1) are located on plasmids, enabling horizontal gene transfer between bacteria of the same and different species [3]. Reports on plasmid-mediated colistin resistance are increasing worldwide, and in June 2016, a second plasmid, *mcr*-2, was described [4]. Plasmids are known for rapid dissemination and, if so, might cause a progression of Enterobacteriaceae from extensive drug resistance to pandrug resistance. Since colistin is widely used in veterinary medicine, it is hypothesised that there might be a connection between colistin-resistant bacteria in water and vegetable samples, and humans [5].

In Switzerland, carbapenem resistance is still rare (under 0.5% for both *Escherichia coli* and *K. pneumoniae* [6]) and consecutive use of colistin low (0.01 defined daily doses (DDD)/1000 inhabitants/day in outpatient settings and 0.3 DDD/100 bed-days in hospitals [7]). However, colistin is commonly used in livestock animals, mainly pigs [8], and invasive infections with colistin-resistant strains have been reported [9]. Given the importance of enhancing knowledge on the breach of this last resort antibiotic, we sought to determine risk factors for human colonisation or infection with colistin-resistant *E. coli* and *K. pneumoniae* in a setting where colistin is mainly used for animal production.

## Methods

### Design, setting and ethics

The University Hospital Basel (USB) is an 855-bed tertiary-care centre with ca 32,000 admissions per year, situated in the city of Basel in Switzerland. The Felix Platter Hospital (FPS) is a 450-bed university-affiliated geriatric and rehabilitation centre with ca 4,500 admissions per year in Basel, Switzerland. The study design is a retrospective case–control study with a matching of one case to three controls. Controls were matched according to the pathogen, the ward type (e.g. ICU, surgical, medical, outpatient setting), the isolation site, and the time of isolation. The study was approved by the local ethics committee (EKNZ BASEC 2016–00832).

### Patients and data collection

During a 5-year period (January 2011 to November 2015), all *E. coli* and *K. pneumoniae* isolates analysed for antibiotic susceptibility to colistin were included as potential cases. The following data was retrospectively collected using the patients’ electronic charts and/or paper charts: demographic information, underlying diseases, Charlson comorbidity index, previous invasive procedures, previous antibiotic treatment, known colonisation with multi-drug resistant bacteria (e.g. meticillin-resistant *Staphylococcus aureus* (MRSA), ESBL), previous stay abroad and hospitalisation abroad, as well as previous hospitalisation. Invasive procedures, medication, hospitalisation and travel abroad during the 3 months before detection of colistin-resistant Enterobacteriaceae were recorded. Infection vs colonisation was defined using the criteria of the United States’ (US) Centers for Disease Control and Prevention [10]. Death due to any cause or death attributable to infection within the same period of hospitalisation or outpatient treatment was assessed.

### Antibiotic susceptibility testing

All samples with growth of Enterobacteriaceae were screened for colistin resistance, which was determined with a microdilution-like technique on an automated VITEK 2 system (bioMérieux Inc., Durham, North Carolina, US) in an ISO/IEC 17025 accredited laboratory with regular and independent quality controls and external auditing. Breakpoints for susceptibility testing were applied in accordance to the European Committee on Antimicrobial Susceptibility Testing (EUCAST) guidelines defining colistin resistance at the minimum inhibitory concentration (MIC) of > 2 mg/L [11]. Additional susceptibility testing was performed according to routine clinical practice using the VITEK 2 system (bioMérieux, Durham, North Carolina, US).

### Statistics

Case–control matching was done using R (version 3.3.0) with the MatchIt software package [12,13]. Matches were identified based on a non-parametrical preprocessing and exact agreement of the submitting ward (outpatient, medical, surgical, ICU (either surgical or medical), gynaecology, acute geriatric and FPS), identified organism (*E. coli* or *K. pneumoniae*), material category (blood, urine, sputum, superficial swab, biopsy, stool or acquired for hospital hygiene) and subsequently on the date the sample was taken. To compare pertinent clinical data between cases and controls, chi-squared and Fisher’s exact test (where appropriate) were used for comparisons of proportions. For continuous variables, the Shapiro-Wilk test was used to distinguish between normal and abnormal distributions. Normally distributed variables were analysed using the Student’s t-test and non-normally distributed variables using the Mann–Whitney U test.

Conditional univariable logistic regression was used to calculate odds radios (OR) for the association of risk factors for colistin resistance. All variables found to be significant in univariable analysis were included in the conditional multivariable regression model. Logistic regression using stepwise forward and backward selection was applied to identify variables which were independently associated with colistin resistance. Sensitivity analysis was performed by excluding all cases with an MIC for colistin of 3 mg/L and their respective controls and repeating the multivariable logistic regression model. P values ≤ 0.05 were considered significant. Statistical analysis was performed with STATA version 12.0 (Stata Corp., College Station, Texas, US).

## Results

**Figure 1:**
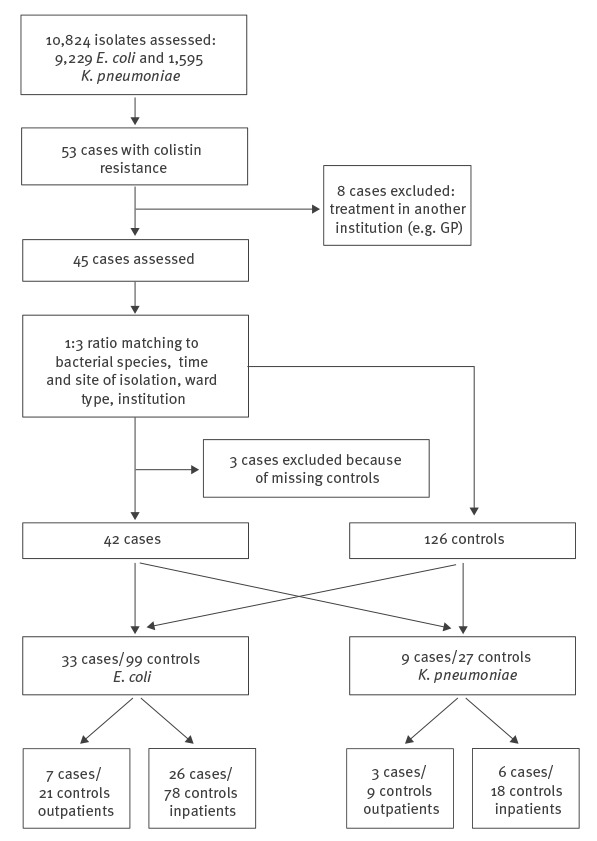
Flowchart of the selection of cases and controls, matched case–control study into risk factors for colistin-resistant Enterobacteriaceae, Switzerland, January 2011–November 2015

**Figure 2:**
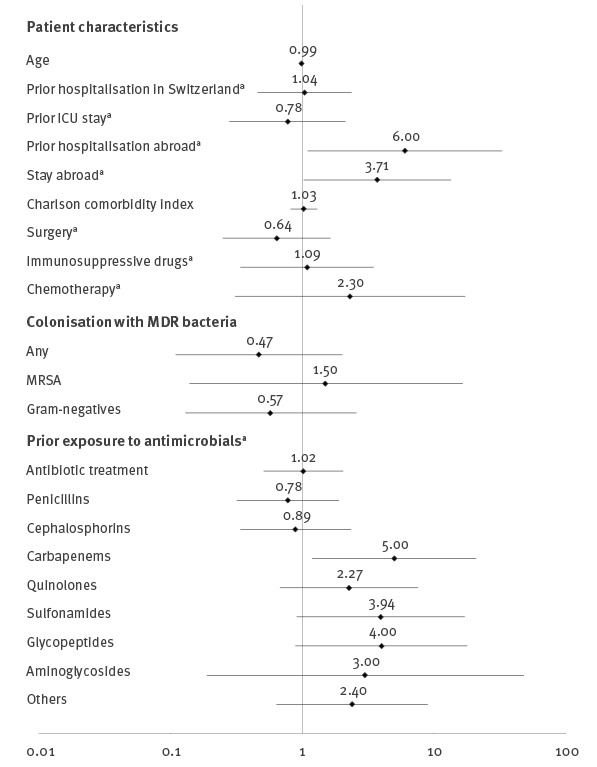
Univariable analysis of risk factors for colistin resistance, matched case–control study into risk factors for colistin-resistant Enterobacteriaceae, Switzerland, January 2011–November 2015

During the study period, a total of 10,824 isolates (9,229 isolates of *E. coli* and 1,595 isolates of *K. pneumoniae*) were analysed for colistin susceptibility. From these, 53 patients were identified with colistin-resistant *E.coli* or *K. pneumoniae*. Eight patients had to be excluded because of missing information due to treatment at other institutions and three patients were excluded due to missing controls. For the final analyses, 42 cases and 126 matched controls were included (Figure 1). Among 42 cases, 33 colistin-resistant *E. coli* and nine colistin-resistant *K. pneumoniae* were identified. MICs ranged from 3 to 48 mg/L (median: 6; interquartile range: 4–12). As shown in the Table, age, sex, Charlson score and underlying diseases did not differ between cases and controls. Infection or colonisation with *E. coli* or *K. pneumoniae* was equally distributed. Outcome defined as death due to any cause or death attributable to infection did not differ. There was no difference between overall exposure to antibiotics between cases and controls, but prior exposure to carbapenems was associated with colistin resistance (Figure 2, Supplementary Table 1). One of the colistin-resistant strains and none of the colistin-susceptible strains were identified as carbapenem-resistant. Prior exposure to colistin was recorded in two cases and none of the controls, but there was no significant association with prior exposure to colistin. Stay abroad and hospitalisation abroad during the prior 3 months was associated with colistin resistance (Figure 2), but only prior exposure to carbapenems remained associated in multivariable analyses (OR: 5.00; 95% confidence interval (95% CI): 1.19–20.92; p = 0.028). Prior hospitalisation in Switzerland was not associated with colistin resistance. Sensitivity analyses confirmed these results (data not shown).

*E. coli: Escherichia coli*; GP: general practitioner; *K. pneumoniae: Klebsiella pneumoniae*.

**Table:** Comparison of demographics, clinical characteristics, exposures, and outcomes between cases and controls, matched case–control study into risk factors for colistin-resistant Enterobacteriaceae, Switzerland, January 2011–November 2015

Characteristic	Cases (n = 42)		Controls (n = 126)		p value
	n/median	% or IQR	n/median	% or IQR	
Age (years)	70	61–84	78	67–84	0.210
Male	30	71.4	86	68.3	0.700
Charlson comorbidity index	2	1–4	2	1–3	0.831
**Inpatients**					
Prior hospitalisation (days)	15	8–28	18	12–37	0.070
Prior ICU days	3	2–6	2	2–5	0.169
**Underlying disease/condition**					
Cardiac disease	14	33.3	52	41.3	NA
Peripheral vascular disease	6	14.3	12	9.5	
Cerebrovascular disease	4	9.5	20	15.9	
Dementia	11	26.2	28	22.2	
Chronic lung disease	10	23.8	19	15.1	
Connective tissue disease	0	0.0	2	1.6	
Peptic ulcer disease	3	7.1	7	5.6	
Hemiplegia	0	0.0	4	3.2	
Chronic renal disease	13	30.9	43	34.1	
Liver disease	2	4.8	4	3.2	
Cancer without metastasis	4	9.5	12	9.5	
Metastatic cancer	3	7.1	10	7.9	
Leukaemia	1	2.4	9	7.1	
Lymphoma	1	2.4	4	3.2	
HIV	1	2.4	2	1.6	
AIDS	0	0.0	2	1.6	
Solid organ transplantation	3	7.1	3	2.4	
HSCT	1	2.4	4	3.2	
**Invasive procedures**					
Surgery before colistin^a^	11	26.2	41	32.5	0.441
**Antibiotic treatment before colistin^a^**	**18**	**42.9**	**52**	**41.9**	
Penicillins	8	19.1	29	23.0	0.917
Cephalosporins	6	14.3	20	15.9	
Carbapenems	5	11.9	3	2.4	
Quinolones	5	11.9	7	5.6	
Sulfonamides	5	11.9	5	4.0	
Macrolides	1	2.4	0	0.0	
Aminoglycosides	1	2.4	1	0.8	
Glycopeptides	4	9.5	3	2.4	
Colistin	2	4.76	0	0.0	
Others	4	9.5	5	4.0	
**Other medication^a^**					
Immunosuppressants	5	11.9	14	11.1	1.000
Chemotherapy	2	4.8	3	2.4	0.600
**Clinical relevance**					
Infection (vs colonisation)	23	54.8	79	62.7	0.362
**Detection site**					
Blood stream	4	9.5	12	9.5	1.000
Urogenital	33	78.6	99	78.6	
Respiratory	1	2.4	3	2.4	
Superficial	1	2.4	4	3.2	
Abdominal	1	2.4	2	1.6	
Screening	2	4.8	6	4.8	
**Treatment**					
No treatment	17	40.5	39	30.2	0.21
**Specific antibiotic exposures**					
Penicillins	5	11.9	21	16.7	0.255
Cephalosporins	7	16.7	17	13.5	
Carbapenems	4	9.5	6	4.8	
Quinolones	4	9.5	19	15.1	
Sulfonamides	0	0	12	9.5	
Macrolides	0	0	0	0	
Aminoglycosides	0	0	0	0	
Glycopeptides	0	0	0	0	
Colistin	0	0	0	0	
Others	2	4.8	5	4.0	
**Outcome**					
Death due to any cause	3	48.6	10	7.9	1.000
Death attributable to infection	0	7.1	6	5.0	0.339

HSCT: hematologic stem cell transplantation; ICU: intensive care unit; IQR: interquartile ratio; NA: not applicable.

^a^ During the 3 months before detection of colistin-resistant Enterobacteriaceae.

ICU: intensive care unit; MDR: multidrug-resistant; MRSA: meticillin-resistant *Staphylococcus aureus*.

^a^ During 3 months prior to detection of colistin-resistant Enterobacteriaceae.

**Figure 3:**
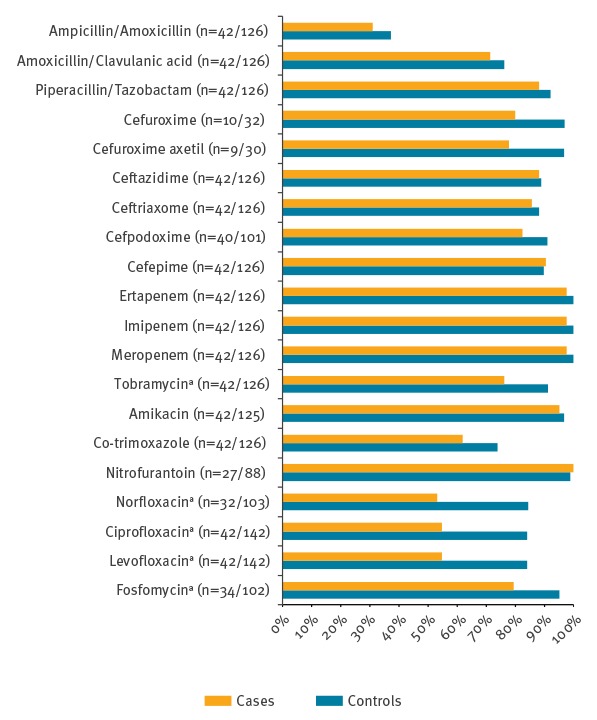
Antibiotic susceptibility testing of cases and controls, matched case–control study into risk factors for colistin-resistant Enterobacteriaceae, Switzerland, January 2011–November 2015

^a^ P < 0.05.

Even though there was no difference in prior exposure to quinolones, fosfomycin and tobramycin, susceptibility testing showed significantly higher resistance rates against these antibiotics in cases as compared with controls (Figure 3, Supplementary Table 2). Proportions of carbapenem resistance on the other hand did not differ between cases and controls.

## Discussion

Overall, colistin resistance occurred in a very low proportion of *E. coli* and *K. pneumoniae* isolates collected during routine clinical practice at two university-affiliated tertiary care centres, compatible with reported low carriage rates of 3.8% in asymptomatic patients in Switzerland [14,15].

Prior exposure to carbapenems was the only identified risk factor for colonisation or infection with colistin-resistant *E. coli* or *K. pneumoniae*. We cannot rule out that the lack of a significant association between hospitalisation abroad or stay abroad and colistin resistance results from the limited sample size included in this study. Prior exposure to colistin was not related to detection of colistin resistance, but only two patients were exposed. Colistin resistance was associated with higher resistance rates to quinolones, fosfomycin and tobramycin, but not carbapenem resistance. Carbapenem-resistant Enterobacteriaceae are known to have concurrent resistance to colistin, fosfomycin and quinolones [16-18], but resistance clusters without concomitant carbapenem resistance have not been described so far and its role and aetiology remains unclear. High rates of colistin resistance in human samples have mainly been reported in countries with high proportions of carbapenem-resistant Gram-negative bacteria and subsequent widespread use of colistin in healthcare settings. In the initial description of the plasmid-mediated colistin resistance mechanism *mcr*-1 in China [3], higher rates of *mcr*-1 carriage were reported in *E. coli* isolates isolated from raw meat and animals (15% and 21%, respectively) than from clinically relevant inpatient samples (1%). These findings suggest that this novel antibiotic resistance mechanism may have evolved primarily in livestock, where exposure to colistin and subsequent selection pressure was highest, and then slowly trickled through and disseminated in humans. More recent reports of prevalence of colistin resistance in livestock differ between animal species and countries, for example between 0% in beef or dairy products and 17.9% in isolates deriving from turkeys in Germany [19], or 0.5% and 5.9% in isolates collected from pigs and turkeys in France, respectively [20]. Even though consumption of colistin in veterinary medicine has decreased in Switzerland in recent years, as well as in other European countries [21,22], it is still widely used with an average consumption of 0.6mg/population corrected unit in Switzerland in 2015 and 0.03 to 10.0mg/kg estimated biomass (average 1.3mg/kg estimated biomass) in 2014 in other European countries [7]. In the latest Joint Interagency Antimicrobial Consumption and Resistance Analysis (JIACRA) report [23], consumption of polymyxins in food-producing animals outweighed reported consumption in humans in most European countries and a significant correlation was found between the use of and resistance to colistin in food-producing animals. Our data point to a source of colistin-resistant strains within Switzerland ­– food-producing animals being a possible source – suggesting that colistin resistance may increase steadily if selected for by increasing use of broad-spectrum antimicrobials without the need for further introduction from high-endemicity settings. We acknowledge, however, that our study provides no evidence to support this hypothesis.

The associations between prior carbapenem exposure, as well as concomitant resistance to quinolones, fosfomycin and tobramycin with colistin resistance identified in our study, possibly suggest that low-grade dissemination of colistin-resistant strains in humans is mainly fueled by selection pressure occurring in patients exposed to different antimicrobials. The lack of associations between hospitalisation abroad and colistin exposure with the identification of colistin-resistant strains points to a source possibly located within Switzerland and not entertained by colistin exposure in patients. Our findings are further supported by detection of *mcr*-1-harbouring, ESBL-producing Enterobacteriaceae in surface water, suggesting environmental contamination [5] and by a report on transferable IncI2 (size ca 60–61 kb) and IncX4 (size ca 33–35 kb) type plasmids, each bearing *mcr*-1, being found to be associated with both human and food isolates in Switzerland, thus providing evidence that the food chain may be an important transmission route for *mcr*-1-bearing plasmids [14].

There are few published reports of clinical risk factors for colistin resistance and studies are mainly from high-endemicity settings for carbapenem resistance or with a limited patient collection (e.g. only critically ill patients, only bloodstream infections) [24,25]. Often, a small number of patients, usually under 100, and retrospective study designs were used. Known risk factors for colistin resistance are previous antibiotic therapy (e.g. colistin, beta-lactam/beta-lactamase inhibitors, carbapenems, glycopeptides), previous hospitalisation and previous colonisation with multidrug-resistant bacteria (e.g. KPC) [26,27]. Wang et al. described male sex, immunosuppression and use of fluoroquinolones as additional risk factors [28]. Our retrospective study has the same limitations as those reports, but adds to the literature as it derives from a low-endemicity setting for carbapenem resistance, which precludes a prospective trial. We acknowledge that the number of cases included in our study is limited, as expected when performing a study in a low-endemicity setting. However, studying risk factors in a low endemicity setting still has its merits, one of them being transferability of our findings to similar settings, as fortunately, many European countries still have low colistin resistance levels such as in Switzerland. Our study provides first clues as to which patients may be primarily affected and how colistin resistance may be introduced into a population in similar settings. Due to the limited sample size, we cannot rule out the lack of a significant association between hospitalisation abroad or stay abroad and colistin resistance. As clinical characteristics were collected by retrospective medical chart review, certain exposures may have been underestimated and we also cannot rule out the potential for residual confounding due to unmeasured variables. To minimise possible confounding, we carefully matched our study patients to controls. To improve generalisability we included patients from two university-affiliated centres. Due to the retrospective nature of our study, the mechanism of the colistin resistance could not be determined, thus we cannot draw any conclusions about whether our results apply to chromosomally mediated resistance or resistance conferred by any of the *mcr*-genes; however, *mcr* plasmids as a mechanism of resistance are very rare in Switzerland. EUCAST has recently issued a warning regarding the susceptibility testing of colistin [29]. The determination of colistin susceptibility using VITEK 2 is discussed controversially; whereas some publications showed a sufficient test performance in *K. pneumoniae* and *Acinetobacter baumannii* isolates to determine the resistance groups [30], others showed categorical agreement in less than 90% with very major error rates of 36% [31]. Of note, in our collection most isolates were clearly susceptible or resistant with estimated MICs of ≤ 2 mg/L or above > 3 mg/L, showing no MICs close to the breakpoint. To further address this concern, we performed a sensitivity analysis excluding all cases with an MIC for colistin of below 4 mg/L and their respective controls.

In conclusion, in a low-endemicity setting for carbapenem resistance, prior exposure to carbapenems was the only risk factor for colonisation or infection with colistin-resistant *E. coli* or *K. pneumoniae*. Prior exposure to colistin was not related to detection of colistin resistance, which mainly occurred in the absence of concurrent carbapenem resistance. Our findings suggest that sources of colistin-resistant Enterobacteriaceae may be present in the absence of widespread colistin use in human medicine and that selection pressure constitutes an important driver for the occurrence of colistin-resistant strains in clinical isolates.
